# Patient and family involvement in Choosing Wisely initiatives: a mixed methods study

**DOI:** 10.1186/s12913-022-07861-2

**Published:** 2022-04-07

**Authors:** Chloe de Grood, Emma E. Sypes, Daniel J. Niven, Fiona Clement, Emily A. FitzGerald, Shelly Kupsch, Shelly King-Hunter, Henry T. Stelfox, Jeanna Parsons Leigh

**Affiliations:** 1 Department of Community Health Sciences, Cumming School of Medicine, University of Calgary, Calgary, Alberta Canada; 2 Department of Critical Care Medicine, Cumming School of Medicine, University of Calgary, Calgary, Alberta Canada; 3 O’Brien Institute of Public Health, Cumming School of Medicine, University of Calgary, Calgary, Alberta Canada; 4 Faculty of Health, School of Health Administration, Dalhousie University, Halifax, Nova Scotia Canada

**Keywords:** Choosing Wisely, Content analysis, Low-value care, Qualitative interviews, Template analysis

## Abstract

**Background:**

Patients are important stakeholders in reducing low-value care, yet mechanisms for optimizing their involvement in low-value care remain unclear. To explore the role of patients in the development and implementation of Choosing Wisely recommendations to reduce low-value care and to assess the likelihood that existing patient resources will change patient health behaviour.

**Methods:**

Three phased mixed-methods study: 1) content analysis of all publicly available Choosing Wisely clinician lists and patient resources from the United States of America and Canada. Quantitative data was summarized with frequencies and free text comments were analyzed with qualitative thematic content analysis; 2) semi-structured telephone interviews with a purposive sample of representatives of professional societies who created Choosing Wisely clinician lists and members of the public (including patients and family members). Interviews were transcribed verbatim, and two researchers conducted qualitative template analysis; 3) evaluation of Choosing Wisely patient resources. Two public partners were identified through the Calgary Critical Care Research Network and independently answered two free text questions “would this change your health behaviour” and “would you discuss this material with a healthcare provider”. Free text data was analyzed by two researchers using thematic content analysis.

**Results:**

From the content analysis of 136 Choosing Wisely clinician lists, six reported patient involvement in their development. From 148 patient resource documents that were mapped onto a conceptual framework (Inform, Activate, Collaborate) 64% described patient engagement at the level of Inform (educating patients). From 19 interviews stakeholder perceptions of patient involvement in reducing low-value care were captured by four themes: 1) impact of perceived power dynamics on the discussion of low-value care in the clinical interaction, 2) how to communicate about low-value care, 3) perceived barriers to patient involvement in reducing low-value care, and 4) suggested strategies to engage patients and families in Choosing Wisely initiatives. In the final phase of work in response to the question “would this change your health behaviour” two patient partners agreed ‘yes’ on 27% of patient resources.

**Conclusions:**

Opportunities exist to increase patient and family participation in initiatives to reduce low-value care.

**Supplementary Information:**

The online version contains supplementary material available at 10.1186/s12913-022-07861-2.

## Background

Low-value care consists of tests, treatments or procedures with little or no benefit to patients, or the cost or potential to cause harm exceeds expected benefits [1]. Low-value care burdens patients and healthcare systems. Estimates suggest that low-value (e.g., overtreatment or care that will not help patients) care may account for up to one-third of wasteful healthcare spending [2–7]. Reductions in low-value care (i.e. de-implementation) may facilitate provision of more effective, higher value care [8, 9]. Several initiatives that aim to reduce low-value care exist. Most notable is the Choosing Wisely campaign, an international effort that began in 2012 to engage care providers and patients in conversations to question low-value care (https://www.choosingwisely.org/). The Choosing Wisely campaign in collaboration with medical societies, produces specialty specific information for clinicians and patients, on tests, treatments and procedures that may be considered low-value. Despite this and other evidence on the burden and harms associated with low-value care, its use persists [3, 10].

Patients are stakeholders in the reduction of low-value care. Previous research related to the prioritization of patient and family-centered care has reported that patients should be meaningfully engaged in all aspects of the healthcare process, including in the planning and execution of research concerning healthcare decision making [11–13]. Studies have also suggested that patients regularly have a desire to be involved in decision making at the health system and unit levels provided that their perspectives are equally valued and consequences from decisions that result from their involvement are explained to them [14]. Previous examination of barriers to de-implementation indicate that patient perceptions and desires are often viewed as a barrier to reducing low-value care [15, 16]. However, a recent knowledge synthesis highlights that engaging patients through education and shared-decisions making significantly decreases the use of low-value care [17]. Perceptions of both patients and healthcare leaders on patient involvement in reducing low-value care are not well understood in the North American context.

The objective of this study was to explore the role of patients and families in the development and implementation of Choosing Wisely recommendations and to assess the likelihood that existing patient resources would change patient behaviour. This included exploring the perceptions of Choosing Wisely list creators and patients and families on the role of patients in Choosing Wisely.

## Methods

### Overall study design

We conducted a three-phase mixed methods study. Phase one consisted of collection and content analysis of all Choosing Wisely clinician lists (list of recommendations, by specialty, that identify tests, treatments and procedures that are considered to be low-value) and patient resources (pamphlets for patients to help them learn about tests, treatments, procedures to question and when they are necessary or not) from the United States and Canada (http://www.choosingwisely.org, https://choosingwiselycanada.org/). Phase two was a qualitative template analysis [18] of semi-structured interviews with patients, families and professional society leaders designed to better understand perceptions of patient and family involvement in Choosing Wisely initiatives and healthcare decision making. Phase three was a review of Choosing Wisely patient resources by patient partners (team members with lived experience in the Canadian healthcare system). University of Calgary Conjoint Health Research Ethics Board approved this study (Ethics ID#: REB18–1272).

### Phase one: collection and analysis of Choosing Wisely clinician lists and patient resources

#### Objective

To describe the current role of patient and family involvement in Choosing Wisely clinician list and patient resource document development in the United States and Canada.

#### Design

The totality of the Choosing Wisely public facing materials, clinician lists and patient resources were downloaded and saved in PDF format from the United States and Canadian Choosing Wisely websites on Feb 10, 2018. Two separate data extraction templates were created, one for clinician lists and one for patient resources due to the different audience of focus for these respective documents. A standardized data extraction template was created to examine the clinician list variables: title, release date, stakeholders involved in list creation, patient population targeted, clinical setting, type of low-value care (test or treatment), and nature of patient involvement [Additional file 1]. If patients participated their involvement was mapped onto a conceptual framework for patient engagement: partner, engage, inform, or empower [Additional file 2] [19]. In a similar fashion to the clinician list extraction form, a separate data extraction form was created for Choosing Wisely Patient Resources to describe where and how patients were involved in their creation and perception of relevance to patients. Data was extracted on the following fields: location, free text description of overall message, defining effect for low-value care (ineffective, cost, risks out-weigh benefits), languages available and Simple Measure of Gobbledygook (SMOG) readability score [Additional file 3] [20, 21]. Patient resources were mapped onto a conceptual framework that described patient engagement as Inform (educating patients), Activate (tools to prompt action of patients) or Collaborate (interaction and engagement of patients and providers) [22]. Each data extraction template was pilot tested on 10 clinician lists and 10 patient resources documents respectively, in duplicate, by two researchers (CD, ES) and refined until a reliability coefficient for binary items of greater than 0.8 was achieved. Disagreement was resolved through discussion between the two researchers.

#### Data collection and analysis

Data extraction was conducted from November 28, 2018 to September 30, 2019. Quantitative data were summarized descriptively using frequencies and percentages within categories. Qualitative data were summarized using thematic content analysis [23, 24]. Two researchers (CD, ES) read and coded all free text responses independently. Researchers met to collapse and condense codes and develop, define, and name themes. Development of themes was an iterative process whereby researchers read and reread through free text responses to search for disparate cases and refine themes as necessary to include all relevant data.

### Phase two: qualitative interviews with representatives of professional societies and patients and families

#### Objective

To describe stakeholder perceptions of patient and family involvement in the development of Choosing Wisely clinician lists and patient resource documents.

#### Participants

We purposively aimed to recruit representatives from 12 professional societies that developed Choosing Wisely clinician lists identified from Phase one (6 that reported, and 6 that did not report, involving patients in clinician list development,) to participate in semi-structured telephone interviews. Leaders of professional societies were recruited by email invitation. Non-responders received three follow-up invitations. We also aimed to recruit 12 patients and family members through social media (e.g., Twitter) and snowball sampling through leaders of professional societies or other patients and families who participated in an interview. Non-traditional routes of recruitment are suggested when attempting to recruit a difficult to reach audience [25, 26]. Specifically, we utilized unpaid outreach with 19 patient organization (any organization focused on patients and caregivers) groups on Twitter who posted or retweeted our recruitment messages to their targeted audiences [Additional file 4]. Interview participants were reminded that interviews were voluntary and that they could end the interview at any time. Interviews continued until thematic saturation was achieved [23].

#### Interview guide

Interviews were semi-structured to allow for full exploration of participants’ experiences. Two interview guides were developed with similar questions, but wording geared towards patients and families in one guide and to professionals who developed Choosing Wisely material in the other [Additional file 5]. Interview questions in both interview guides explored participant perceptions of physicians and patients and family members having conversations about low-value tests and treatments, what the future role of patients and families in the removal of low-value care should look like, and perceived benefits and risks to inclusion of this stakeholder group in this process. The interview guide for patients and families was pilot tested with patient partners to address interviewer bias, leading and ambiguity in interview questions [27]. The interview guide created for representatives of professional societies was pilot tested with two individuals who had experience with reducing low-value care in a medical leadership role. These individuals were identified through personal contacts. Field testing was conducted to ensure that relevance, order and flow of the questions were appropriate to elicit perceptions on involvement of patients and families in identifying low-value care for de-implementation [27].

#### Data collection and analysis

Qualitative semi-structured interviews were conducted after obtaining informed consent from participants. Interviews were conducted by telephone in a private office by one researcher (CD) between March 19, 2019 and July 11, 2019. Participant demographics were collected with a short verbally administered questionnaire at the end of each interview [Additional file 6]. Demographic information was stored in a separate password protected database and participants were assigned a unique identifier. Interviews were transcribed and deidentified by removal of location, professional organization, and names by an experienced qualitative researcher (CD). Data analysis followed a template analysis method [23] and occurred between March 30, 2019 and August 5, 2019 on all transcripts in six iterative steps: 1) Familiarization with the data through a close reading by both researchers (CD, ES) 2) Preliminary coding of the data by each researcher (CD, ES) to identify in NVivo parts of the transcripts that furthered understanding relevant to the research question 3) Organization of initial themes into hierarchical relationships 4) Definition of an initial coding template by both researchers (CD,ES) 5) Application of the coding template to further data and refinement the coding template and 6) Finalization of the template and application to the entire set of transcripts. More detail on analysis can be found in Additional file 7. The final template can be found in Additional file 8.

### Phase 3: review of Choosing Wisely patient resources

#### Objective

To evaluate patient partners’ perceptions of Choosing Wisely patient resource documents.

#### Design

Two patient partners (SL, SK) were invited through the Calgary Critical Care Research Network (https://cumming.ucalgary.ca/research/calgary-critical-care-research-network) to each independently review the totality of Choosing Wisely patient resource documents (*n* = 148) from the USA and Canadian Choosing Wisely websites. Patient partners were selected based on their expertise with lived experience in the healthcare system as well as both having experience conducting research. In their review of each document, patient partners were asked to respond to two binary (yes/no) questions [24, 27]: 1) Will this document change your behaviour? and 2) Would you discuss the material from this document with a healthcare provider (e.g., physician)? For each of the questions the patient partners were asked to provide a brief free text explanation to justify their answer.

#### Data collection and analysis

Patient partners piloted the two questions with 10 randomly selected patient resources documents (5 from USA and 5 from Canada). From the pilot process, the instructions for the patient partners were refined to answer the two questions (and free text justification of their answers) as it related to their own healthcare decision making or the healthcare decision making of a family member or friend. The data collection proceeded with patient partners independently reviewing the remaining 138 patient resource documents between August 24, 2020 – February 1, 2021. Quantitative data from the two yes/no questions was summarized using descriptive statistics in Microsoft Excel frequencies (percent) or means (standard deviation) to summarize responses to binary answer questions. Qualitative data from the free text explanation justifying their yes/no responses was analyzed using thematic content analysis [23]. Two researchers (CD, EF) familiarized with the free text response data and then generated a list of initial codes related to the research questions of behaviour change and intention to discuss with healthcare providers. Together they developed an initial codebook which was applied by each researcher independently to the free text data set in NVivo. After which the researchers met again to review existing codes, add new codes, and collapse redundant codes. Using the refined codebook, the data was recoded in duplicate. Researchers went through two cycles of independently searching for themes and coming together in an analysis meeting to develop, name and define themes.

## Results

### Phase one: content analysis

#### Clinician list creation

Nearly three quarters of the 136 clinician lists were from the United States, and a third (31%) were released in 2017 (Table 1). Most (70%) of the clinician lists did not specify a target clinical population. Only 6 (5%) of the clinician lists reported involving patients in the development of the list. The six societies that involved patients did so at the partner level (partnerships with patient organizations to develop and implement campaign materials and initiatives) ^19^ framework and involved anywhere from 1 to 6 individuals [Additional file 9]. Patients were involved either as members of the working group that prioritized and selected recommendations for inclusion in the clinician lists or as reviewers of language and content in the final lists.

**Table 1:** Characteristics of North American (USA, Canada) Choosing Wisely Society Lists (*n* = 136)

Characteristics	N (%)
**Country**	
USA	91 (67)
Canada	45 (33)
**Date list released**	
2012	9 (7)
2013	36 (26)
2014	18 (13)
2015	11 (8)
2016	5 (4)
2017	42 (31)
2018	15 (11)
**Population**	
Adult	14 (10)
Pediatric	12 (9)
Not specified	95 (70)
Both	15 (11)
**Focus of recommendations**	
Tests	20 (15)
Treatment	12 (9)
Both	104 (76)
**Methods to develop list published**	
Yes	5 (4)
No	131 (96)
**Patients and/or family members reported to be involved in creation/development of list**	
Yes	6 (5)
No	130 (95)
**Society lists that include patients and/or family members as subject in the recommendations**^**a**^	
Yes	30 (22)
No	106 (78)

^a^Recommendations within the clinician lists that involve patients (e.g., Canadian Critical Care Society– Don’t start or continue life supporting interventions unless they are consistent with the patients’ values and realistic goals of care)

#### Patient resources

We identified 148 Choosing Wisely patient resource documents representing 84 professional societies. On average the SMOG readability score of the 148 patient resources was Grade 9 (+/− 1.8). Over three quarters (76%) of the patient resources were from the United States and were available in two languages (70%) [Additional file 10]. In terms of the overall message of the patient resource documents, most (72%) dealt with the topic of “circumstantial use” (i.e., occasions when certain tests, treatments and procedures may or may not be appropriate). Over a third (39%) mentioned the combined defining effects of low-value care as lack of efficacy, not cost effective and risks outweigh benefits. Over half (64%) of the resources were categorized as inform (resources that provided knowledge), (28%) as inform and activate (resources that educated and prompted action) and (7%) as inform, activate, and collaborate (resources that prompted interaction between patients and providers).

### Phase two: qualitative interviews with representatives of professional societies and patients and families

We invited representatives from 47 professional societies in the United States and Canada to participate in a semi-structured telephone interview. Of these, four declined an interview, and 35 did not respond despite three follow-up attempts. Three representatives from professional societies that involved patients, and five representatives from professional societies that did not involve patients participated in interviews. These included seven physicians and one nurse. We invited 19 Twitter accounts that spanned patient organizations in the United States and Canada to retweet our recruitment tweet, 8 of these organizations retweeted, an additional three organizations and 22 individuals retweeted. From this Tweet there were 10, 521 impressions (times people read the recruitment information on Twitter) and 268 total engagements (times people interacted with this Tweet). From Twitter, 12 patients and families reached out to participate in an interview, of which three never responded to scheduling, despite two reminders. Ultimately nine patients and family members participated in an interview [Additional file 11]. Further recruitment through Twitter was not needed as thematic saturation was achieved following the third iteration of the template.

**Fig. 1:**
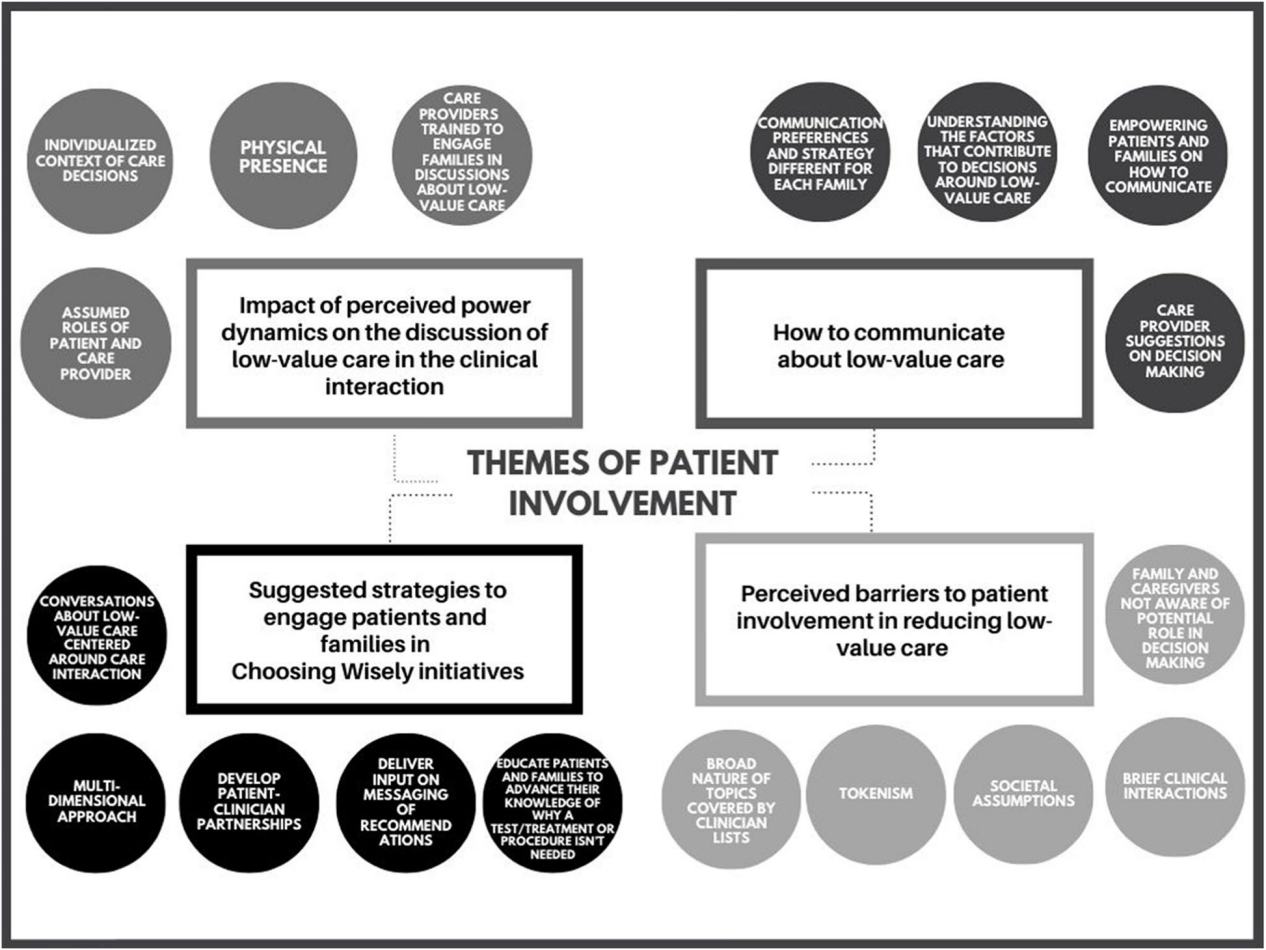
Themes of patient involvement from qualitative interviews

Analysis revealed four overarching themes describing perspectives on present and future patient and family involvement in healthcare decision making regarding low value care: 1) Impact of perceived power dynamics on the discussion of low-value care in the clinical interaction**,** 2) how to communicate about low-value care, 3) perceived barriers to patient involvement reducing low-value care, and 4) suggested strategies to engage patients and families in Choosing Wisely initiatives (Table 2 and Fig. 1). Overarching themes spanned participant groups, but were also comprised of more nuanced subthemes that offered diverse perspectives and characterized thematic tensions between society leaders and patients. Table 2 displays exemplar quotations for all themes and subthemes by participant group.

**Table 2:** Patients and Families (P) and representatives of professional societies’(R) perspectives on patient involvement in Choosing Wisely initiatives (*n* = 17)

Theme and Subtheme	Participant Group	Exemplar Quotation
**Theme 1: Impact of perceived power dynamics on the discussion of low-value care in the clinical interaction**		
Assumed roles of patient and care provider	P/R	“*We need to remove fear, and that power dynamic that exists, that automatic power dynamic that exists between clinicians and providers and physicians to make patients and families not be afraid to ask questions.”* – Interview 3 (Patient)
Individualized context of care decisions	P/R	“*Part of it [care decisions] is always determining what is the value of a test. So if sometimes, it’s the path of least resistance for some people and others they actually value the test even if it’s a negative test … how it is done is a difficult question to answer because I think it really depends on the scenario*.”-Interview 6 (Representative of society)
Physical presence	P	“*I was expected to make decisions but it was relayed through my grandma or a nurse because if I missed when he [the doctor] was there it was a lack of opportunity from a care team perspective*.” – Interview 2 (Patient)
Care providers should be trained to engage families in discussions about low-value care	P	“*Providers probably need to be trained in some ways of having those conversations because I have heard it’s easier to just write a prescription than it is to have a conversation … especially if it’s a patient who has waited two hours at a walk in and is wanting to walk away with a prescription for antibiotics and they are going to be pissed off by the fact that they can’t get that.”* –Interview 3 (Patient)
**Theme 2: How to communicate about low-value care**		
Understanding the factors that contribute to decisions around low-value care	P/R	“*It’s really important for patients to be better educated when there is some type of test, what would the test show and what would we do with that, lots of patients think you have a test and it’s a black and white yes or no and then we have a magic pill to treat you*.”-Interview 16 (Patient)
Communication preferences and strategy different for each family	P	“*If I had questions I called my family physician … because I have a great rapport with my family physician I can do that. So I don’t have a problem to phone and say okay I am not clear on this, what does this mean?”* – Interview 13 (Patient)
Empowering patients and families on how to communicate	P	“*Maybe the doctors need to lead the way because as a patient I would like to be more involved in decision making and empowered in that regard I don’t have the expertise to decide what is unnecessary and what is not.”* -Interview 16 (Patient)
Care provider suggestions on decision making	R	“*Go down Dr. Google and figure things out themselves but I don’t personally see that as a bad thing because I think, you can go and do your research and get a sense of the questions you want to ask instead of kind of being just a pawn going through your care.”* - Interview 9 (Representative of society)
**Theme 3: Perceived barriers to patient involvement in reducing low-value care**		
Brief Clinical Interactions	P/R	“*The short interaction time frame, often times you don’t have a lot of time to you know get the big picture … so a readers’ digest version of your rationale as to why this shouldn’t happen right now [is needed].”* – Interview 2 (Patient)
Societal Assumptions	P/R	“*The lack of a test, does not necessarily mean a lower standard of care, whereas that’s a bit of a misnomer in that people might think it is … those conversations need to be had so that people don’t think that lack of testing equals lack of quality in care*” –Interview 5 (Representative of society)
Family and caregivers may not be aware of potential role in decision making	P	“*I think I was adequately involved because I knew that I could be and I asked for that but I think my family needed to be more educated*.”-Interview 2 (Patient)
Tokenism	R	“*I think we need to figure out how to move beyond that [tokenism], patients have a great depth of information as it relates to their illness … And so training patients to be the voice of the mass of patients and then training providers in how to respectfully engage patients in the process to make it meaningful for everybody.”-* Interview 11 (Representative of society)
Broad nature of topics covered by clinician lists	R	*“If you have a group of recommendations that you can narrow down to a focused groups of patients that would be easier because if we had said we have got this list of 22 candidate recommendations and then farmed them out to all [area of care] patients in the country I just don’t know how you would do that.”* Interview 10 (Representative of society)
**Theme 4: Suggested strategies to engage patients and families in Choosing Wisely initiatives**		
Conversations about low-value care centered around care interaction	P/R	[In response to how should conversations occur] *“Probably at a primary care or when you are receiving care from a physician before you are referred to tests.”-*Interview 15 (Patient)
Consistency in patient engagement	R	“*Its not one discipline who has that responsibility or who has that role, this is inter [disiciplinary], and the patient and the family and the community expect us to be working collaboratively and to be maxing out the knowledge and skill and contribution of each of those disciplines across our practices so I think we are actually needing something that patients and families and communities already expect*” –Interview 10 (Representative of society)
Multidimensional approach	R	*“We have to train individual people, the public through social media, and train the policy makers, we have to train the practitioners and the nurses, it has to be a multipronged approach, there is no way there will be one single easy solution.”* – Interview 11 (Representative of society)
Educate patients and families to advance their knowledge of why a test/treatment or procedures isn’t needed	R	“*Patients’ involvement could be one of requesting additional education about whether a test they have learned about would be useful for them. Then there is discussion to revolve around either the importance or optionality of having that test or doing different tests instead*.” -Interview 18 (Representative of society)
Deliver input on messaging of recommendations	P/R	*“When we had narrowed down the list if we had involved patients in some of the wording or perhaps in speaking to the harms that are not apparent, so [if] we tried to word that in a way that was appropriate and sensitive that would have been a very good opportunity to have engaged a patient to sort of see their perception of our perception of the harm.”*- Interview 9 (Representative of society)
Develop patient-clinician partnerships	P	*“If I had questions I can call my family physician, because I have a great rapport with my family physician I can do that. I don’t have a problem to phone and say okay I am not clear on this, what does this mean?”-*Interview 5 (Patient)

#### Overarching themes

##### Impact of perceived power dynamics on the discussion of low-value care in the clinical interaction

Both patients and representatives of professional societies discussed how low-value care was discussed in the clinical interaction and described the impact of power dynamics on this discussion (Table 2). For example, participants described assumed roles of patient and care provider (e.g., clinician is the knowledge holder, patient is the knowledge receiver) and the individualized context of care decisions (i.e., approach to treatment, planning and engagement varies person to person) as important aspects of what makes a test, treatment or procedure in the context of care decisions in the clinical interaction low-value.

When impacts on understanding of low-value care were stratified by participant group, we found that patients and families emphasized the importance of physical presence (e.g., being in the clinical setting in contrast to reading on their own) for understanding the necessity, or lack thereof, of tests or treatments (Table 2). Patients and family participants also uniquely indicated that their relationship with the care team (e.g., good rapport with clinicians), and care providers who are trained to engage families in discussions around care options and factors in decision-making as an important impact on their understanding of low-value care.

##### How to communicate about low-value care

Both patients and representatives of professional societies highlighted the need to communicate the factors that contribute to decisions around reducing low-value care (e.g., why the test, treatment or procedure may not be needed as well as the potential for associated risks).

When stratified by participant group, patient participants expressed that individual families may have different preferences (e.g., one family member as communicator vs multiple family members and patient) and require different strategies (e.g., speak with the doctor or nurse one on one vs receiving an information pamphlet from a clinic) for communication about low-value care. Participants in the patient and family group also uniquely mentioned the need to feel empowered by providers to communicate and participate in low-value care conversations. In contrast, representatives of professional groups indicated that enabling patients to understand the alternative plan (which may be no test, treatment, or procedure) is key information to include in conversations about low-value care.

##### Perceived barriers to patient involvement in reducing low-value care

During their interviews, participants identified perceived barriers (i.e., factors that hinder participation) to patient involvement in Choosing Wisely campaigns. Prevalent subthemes described across all participants, included: brief clinical interactions; societal assumptions (e.g., not receiving a test or treatment suggests a lower standard of care). Both subthemes impact the ability to engage in conversations about low-value care (Table 2).

While participants from both groups largely reported similar and overlapping barriers, key differences were also present (Table 2). For example, patients readily postulated the lack of a role for patients in decision making as a barrier to their involvement (a subtheme not strongly suggested by representatives of professional societies) (Table 2). In contrast, representatives of professional societies more readily emphasized tokenism (e.g., one patient on a committee dominated by society leaders and clinicians) and the broad nature of topics covered by recommendations (e.g., recommendations span a variety of patient populations and health topics, of which patients may not be familiar) as leading barriers to patient involvement in development of Choosing Wisely recommendations.

##### Suggested strategies to engage patients and families in Choosing Wisely initiatives

Participants also described how patients and families could be engaged in Choosing Wisely recommendations about low-value care as follows: 1) conversations should be centered around the care interaction (i.e., occur at a doctor appointment); 2) consistency in how patients are engaged across professional societies (e.g., via guidelines); 3) multidimensional approach to ensure a representative and appropriately diverse group of patients are involved (e.g., diverse group that are reflective of the population the practices apply to and reaching out to different patients who are involved in this work already and those who are not); 4) delivering input on messaging of recommendations (e.g., rewording to ensure accessibility of language); 5) educate patients and families to advance their understanding of why a test/treatment/procedure is or isn’t needed; 6) develop patient-clinician partnerships to support patient and family involvement.

When the data were stratified by societies that involved patients in developing Choosing Wisely recommendations and societies that did not, representatives of societies that had involved patients indicated that involving patients in identifying low-value practices was an important method of engagement in developing their list of existing clinical practices that should be questioned. Participation could involve membership on committees for identifying low-value care (e.g., members who rank which recommendations addressing low-value care are most important). They suggested benefits of including patient voices in their approach included increased diversity of perspectives and acceptability in reduction of low-value care to patients. Notably, while representatives of societies that did not involve patients in list development were open to involving patients in the future, they spoke of a need to better understand how to involve them in this area of work. In particular, there was mention that patients are not best posed to evaluate the evidence needed to discern what tests, treatments and procedures are low-value.

### Phase three: patient resource review

**Fig. 2:**
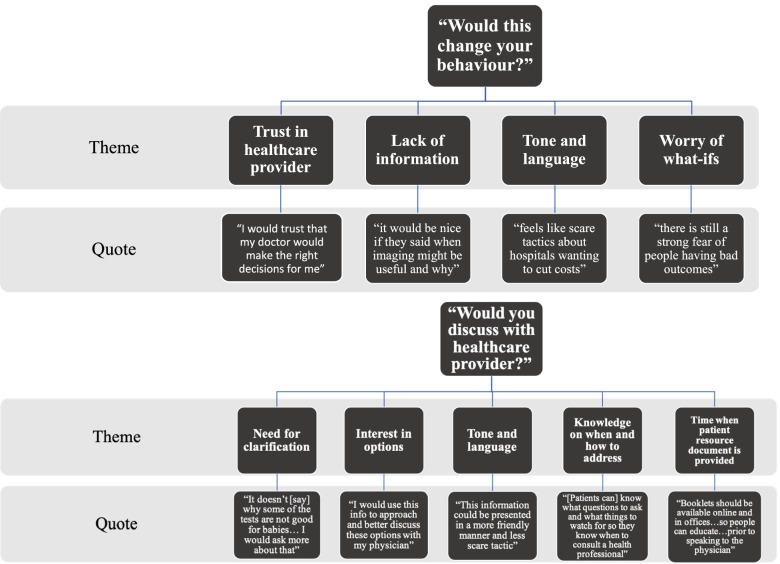
Patient partner review themes and supporting quotations. Patient partner review themes with supporting quotations

Two patient partners each independently reviewed the totality (*n* = 148) of Choosing Wisely patient resource documents. In response to question 1 “would this change your health behaviour” patient partners agreed ‘yes’ on 27% of the documents. When asked question 2 “would you discuss this material with a healthcare provider” patient partners agreed ‘yes’ on 58% of documents. Qualitative thematic content analysis of free text justifications to each of these ‘yes’ or ‘no’ questions yielded overarching themes (Fig. 2). Themes related to question 1 included: i) Trust in healthcare provider decisions and opinions (e.g., providers are up to date on best evidence); ii) Lack of information (e.g., more information is needed in documents to highlight pros and cons); iii) Tone and language of information (e.g., need to be tailored and accessible to patients and families); and iv) Worry of ‘what ifs’ (e.g., something may be missed by skipping a test). Themes related to question 2 included: i) Need for clarification (e.g., information can be brought to clarify with healthcare providers); ii) Interest in options (e.g., information is there to help discuss other choices); iii) Tone and language impact intentions (e.g., language undermines healthcare provider); iv) Knowledge on when and how to address (e.g., guides what content to discuss); v) Timing when patient resource document is provided (e.g., educate patient in order to have discussions). Exemplar quotations can be found in [Additional file 12].

## Discussion

A growing number of initiatives [10, 28–30], including the Choosing Wisely [31] campaign exist to reduce low-value care. These initiatives often involve partnerships with clinical societies, patients and patient groups [19], however recent literature suggests that patient expectations are often a barrier to de-implementation [15, 16], while other research emphasizes patient focused education and shared decision making with physicians as a facilitator of decreases in the use of low-value care [17]. As such, it how best to integrate patients into the process of reducing low-value care remains unclear as there is little consensus currently on the role that patients should assume [32]. Using a mixed-methods approach, our study identified 136 Choosing Wisely clinician lists, among which 6 involved patients in list development. Of these, all did so by including patients as embedded members of the working group that prioritized and selected recommendations or as reviewers of content in the lists. We interviewed 17 stakeholders and identified four themes describing perspectives on present and future patient and family involvement in healthcare decision making regarding the removal of low value care. Two patient partners also reviewed 148 Choosing Wisely patient resource documents and agreed ‘yes’ this document would change their health behaviour on 27% of documents and agreed ‘yes’ they would discuss content with a healthcare provider on 58% of documents.

Choosing Wisely is an initiative that was designed to further conversation between clinicians and patients around tests, treatments and procedures that are not needed [31]. Other research indicates that patient involvement in all aspects of health care decision making is important as they are key consumers [11–13] that often would like to be involved in decision making about their own care [14]. A recent systematic review indicated that shared decision making and patient education make a difference in the use of low-value care [17]. Despite this, our study found patients are infrequently involved in the development of Choosing Wisely clinician lists. Furthermore, when patients were involved, it was at the partner level of patient engagement only (i.e., educating patients). Reasons for lack of patient involvement are likely many and complex. In our study, interview participants reported that the brief clinical interaction and societal assumptions (e.g., more care is better care) are key barriers to patient and family involvement in reducing low-value care. Yet other evidence indicates that low-value care may persist due to practices being complex and often include cultural and organizational factors such as perceived patient demand for tests [2, 16, 28, 30, 33], leaders with strong clinical preferences and a lack of clinical practice guidelines [34]. This is further complicated because appropriateness of care is often dependent on characteristics of the population for which the test, treatment or procedure is intended [35, 36]. Limited engagement may also be due to the factors mentioned about complexity of the topic or perceived barriers to engagement such as increased resources (e.g., time), tokenism (e.g., involving one patient to check a box indicating they were engaged) and the possibility of shifting the conversation away from the original agenda [13]. Importantly, our findings indicate that the tone and wording of documents aimed at helping patients understand low-value care in the Choosing Wisely campaign negatively impacted their willingness to change behaviour, as well as their comfort in discussing the topic with a healthcare provider. Review of such documents by people with lived experience in the healthcare system could improve patient acceptability. This aligns with other research indicating that while patients may initially believe that more care is better, when presented with simple information about overuse of tests and procedures that have no benefit, patients questioned their beliefs [37].

In our study patients reported the need to feel empowered to communicate and participate in low-value care conversations. Patient partners indicated the need to be provided the right information at the right time to empower changes. Participants clearly indicated an expectation to be provided with appropriate (in terms of level, tone and language and completeness) information on low-value care. This equipped individuals for dialogue with physicians to clarify and investigate alternative options. In particular, interview participants emphasized the need for healthcare providers to communicate an alternative plan that included no test, treatment or procedure as key information in their discussions. Patient partners also expressed concern over ‘what ifs’ and worry of missing something if no test, treatment or procedure was performed. This offers insight into what is needed in a discussion about low-value care from both patient and healthcare provider perspectives. Our study also found that power dynamics between patients and providers were an important influencer on understandings of low-value care. Existing literature suggests that a logical way to navigate this conversation is through the principles of shared decision making to address understandings of low-value care and fully engage patients in healthcare decision making in the clinical encounter [38–40]. One study using “Five Choosing Wisely questions” (a tool aimed to enable patients to participate in shared decision making and address power imbalances in the clinical encounter) found that patients were interested in understanding low-value care as it pertained to why they felt unwell but did not feel they needed to know more about other areas of low-value care [41]. Other work that has examined physician perceptions of Choosing Wisely initiatives indicated support for this approach as the conversation could be physician led, grounded in the clinical conversation that already occurs and provide a simple message for patients that allows for shared decision making [42]. Despite this, existing evidence also shows that some patients prefer their physician to make decisions that are in their best interest and feel it is not as important for patients to bring forward the idea of questioning care [41]. This stance was reflected in part by patient partners in our study who indicated that they trust the decisions of their healthcare provider and expect them to be up to date on best evidence. Given the variation in stances toward low-value care conversations (e.g. one patient’s desire to be an informed member of their own care team vs. another’s desire to trust fully in their physician’s knowledge and authority), indicates that further research to investigate how best to negotiate the spectrum of shared decision making around low-value care is warranted.

This study is not without limitations. Participants may have been motivated to interview because of mostly positive or negative experiences. Non-participants may have been deterred due to the same reasons. Thus it is possible that important perspectives were missed. In addition, opinions of participants do not necessarily represent all the opinions of a large diverse group such as citizens. To address this, we purposively sought perceptions from different locations, sexes, and ages. Strengths of this study include engagement of patient partners as team members and a mixed methods approach with data collected using three different methods. The use of semi-structured interview format which allows for participants perceptions to be fully explored. This area could benefit from further research, including co-design, by bringing together Choosing Wisely leaders, researchers and patients, with a strategy or framework promoting patient engagement.

## Conclusions

Patients are key consumers of healthcare and targets of Choosing Wisely initiatives, yet they have been infrequently involved in the development of Choosing Wisely clinician lists. Study participants identified four overarching themes related to patient participation in low-value care conversations: 1) Impact of perceived power dynamics, 2) How to communicate about low-value care, 3) Perceived barriers to patient involvement in reducing low-value care; and 4) Suggested strategies to engage patients and families in Choosing Wisely initiatives. Patients should be engaged, when appropriate, in conversations centered around the clinical interaction, and should be involved consistently across professional societies using a multidimensional approach targeted at diverse individuals. Involvement could include input on messaging of recommendations and in some instances membership on committees that select and identify low-value care practices where lived experience could be contributed.

## Supplementary Information

**Additional file 1.** Choosing Wisely clinician list data extraction template.

**Additional file 2.** Born et al. framework for patient engagement in Choosing Wisely campaign.

**Additional file 3.** Choosing Wisely patient resource standard data extraction template.

**Additional file 4.** Recruitment Message. Social media recruitment message.

**Additional file 5.** Interview Guides. Interview guides for patients and families and society member involved in list creation.

**Additional file 6.** Structured Questions. Structured questions from qualitative interviews.

**Additional file 7.** Detailed Description of Data Analysis. Detailed description of data analysis for qualitative interviews.

**Additional file 8.** Final Template.

**Additional file 9.** Characteristics of North American (USA, Canada) Choosing Wisely clinician lists that involved patients and/or family members (*n* = 6).

**Additional file 10.** North American (United States, Canada) Choosing Wisely patient resources focused on reducing low-value care (*n* = 148).

**Additional file 11.** Interview Participant Characteristics.

**Additional file 12.** Patient partner review theme description and supporting free text responses.

## Data Availability

The datasets generated and analysed during the current study are not publicly available due to the small number of professional societies who involved patients in their clinician list development and raw interview transcripts when considered as a whole may potentially be identifying. The data set is available from the corresponding author upon reasonable request.

## Abbreviations

SMOG: Simple Measure of Gobbledygook
USA: United States of America
