# Supplementary material for: Patient and family involvement in Choosing Wisely initiatives: a mixed methods study
Source: BMC Health Serv Res. 2022 Apr 7;22:457. doi: 10.1186/s12913-022-07861-2 (PMC8991491; doi:10.1186/s12913-022-07861-2)
Supplement: Supplementary file 1 — Additional file 1. Choosing Wisely clinician list data extraction template. [file 12913_2022_7861_MOESM1_ESM.docx]

Additional File 1 – Choosing Wisely clinician list data extraction template

| List Title | Date list was released (year) | Were patients in creation/  development of list? (1=Yes, 0=No) | Free text description of patient involvement | How many patients involved? | Born Framework for patient engagement (partner, engage, inform, empower) | Type of low-value care (test, treatment, both) | Clinical Setting (inpatient, outpatient, both, unclear) | Patient population targeted (adult, pediatric, both, unclear) |
| --- | --- | --- | --- | --- | --- | --- | --- | --- |
|  |  |  |  |  |  |  |  |  |
