# Supplementary figures and images for: Patient and family involvement in Choosing Wisely initiatives: a mixed methods study
Source: BMC Health Serv Res. 2022 Apr 7;22:457. doi: 10.1186/s12913-022-07861-2 (PMC8991491; doi:10.1186/s12913-022-07861-2)

Additional File 2 - Born et al. framework for patient engagement in Choosing Wisely campaign.


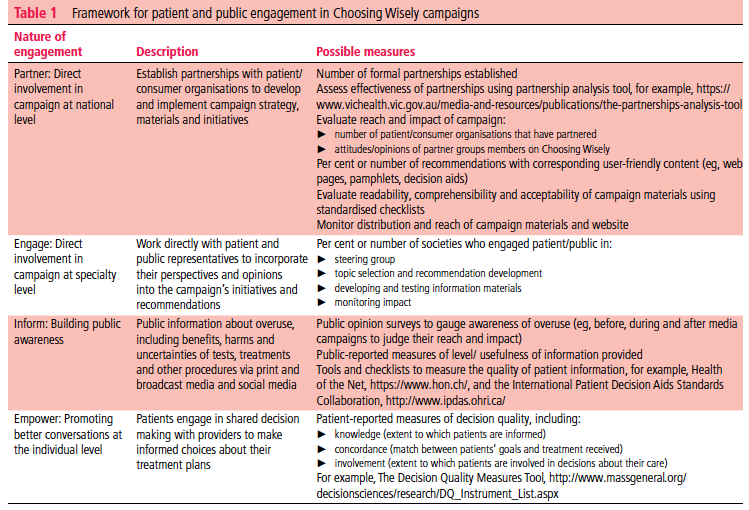

Supplement: Supplementary file 2 — Additional file 2. Born et al. framework for patient engagement in Choosing Wisely campaign. [file 12913_2022_7861_MOESM2_ESM.docx]

Additional File 4 – Recruitment Message


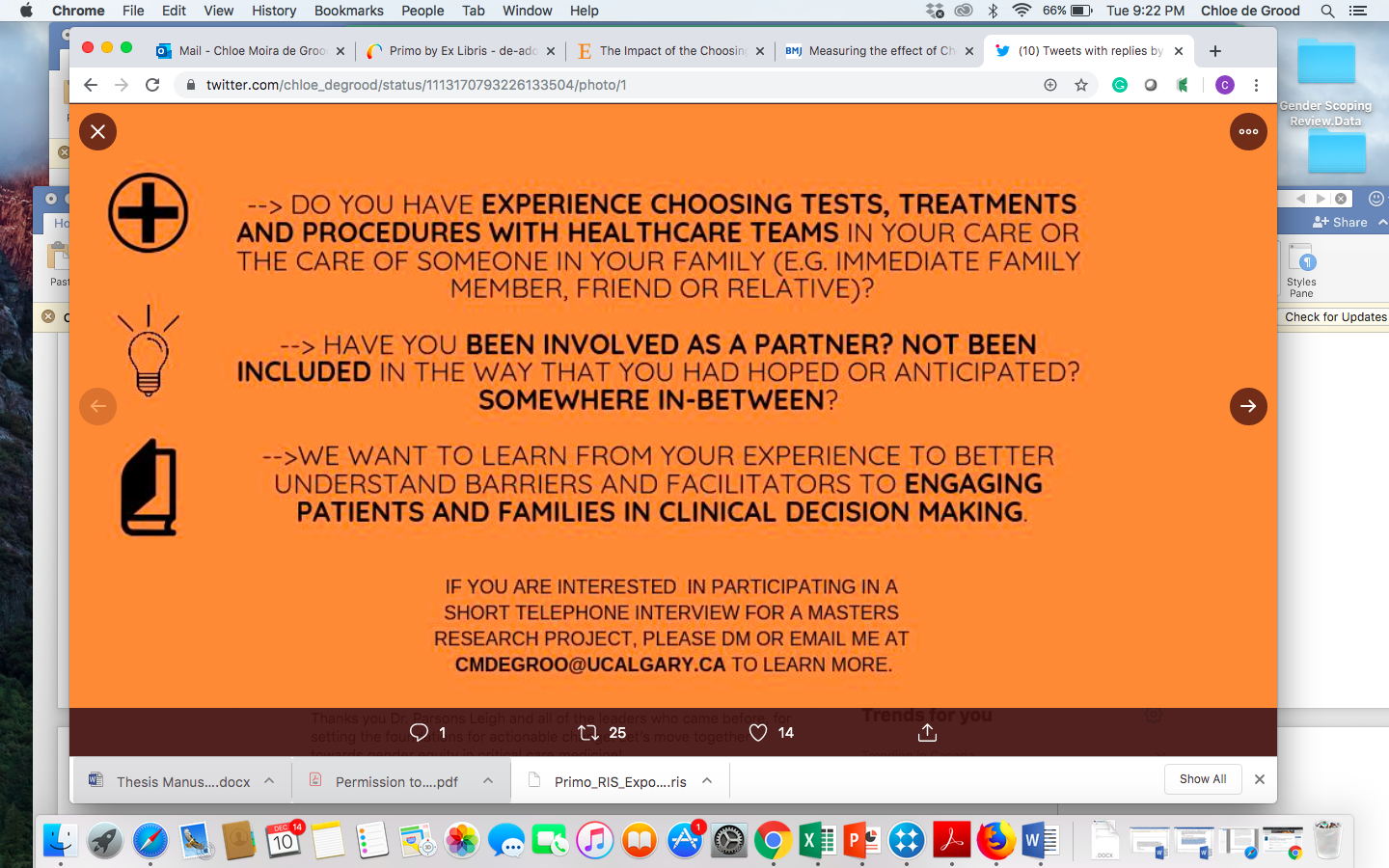

Supplement: Supplementary file 4 — Additional file 4. Recruitment Message. Social media recruitment message. [file 12913_2022_7861_MOESM4_ESM.docx]
