# Supplementary material for: Patient and family involvement in Choosing Wisely initiatives: a mixed methods study
Source: BMC Health Serv Res. 2022 Apr 7;22:457. doi: 10.1186/s12913-022-07861-2 (PMC8991491; doi:10.1186/s12913-022-07861-2)
Supplement: Supplementary file 3 — Additional file 3. Choosing Wisely patient resource standard data extraction template. [file 12913_2022_7861_MOESM3_ESM.docx]

Additional File 3 – Choosing Wisely patient resource standard data extraction template

| Document Title | Location (CAN, USA) | Fiest et al. framework (Inform, Activate, Collaborate) | Free Text justification of framework | SMOG Score | Free text overall messaging | Free text type of low-value care | Languages Available (English, French, Spanish) |
| --- | --- | --- | --- | --- | --- | --- | --- |
|  |  |  |  |  |  |  |  |
