# Supplementary material for: Patient and family involvement in Choosing Wisely initiatives: a mixed methods study
Source: BMC Health Serv Res. 2022 Apr 7;22:457. doi: 10.1186/s12913-022-07861-2 (PMC8991491; doi:10.1186/s12913-022-07861-2)
Supplement: Supplementary file 5 — Additional file 5. Interview Guides. Interview guides for patients and families and society member involved in list creation. [file 12913_2022_7861_MOESM5_ESM.docx]

Additional File 5 - Interview Guides

Interview Guide – Patients and Families

**Preamble:**

Thank you for agreeing to speak with us today. This study is about understanding the role of patients (e.g., family members, relatives, friends) in healthcare decision making. We have asked you to take part in this interview because you have previous experience with healthcare decision making for yourself or your family member, friend or relative.

Choosing Wisely is an international campaign designed to engage care providers and patients in discussions around unnecessary tests, treatments, and procedures. This interview will focus on your previous experiences with making healthcare decisions and your personal perceptions on engaging in discussion with physicians about [your/your family members] care. Our primary focus is to explore the role of patients and family members in healthcare decision making, should they be involved and if so how. We have a series of questions we will ask you. These topics serve as a guide only. If there are other insights you would like to offer, we would like to hear them. We will also ask you a few brief demographic questions. We look forward to the opportunity to learn from your insights.

This interview is completely voluntary. If at any point you feel uncomfortable with the process and wish to end the call, you are free to do so. We recognize that this might be a sensitive topic for you, and we want to make you feel as comfortable as possible. If you feel discomfort and would like to skip a question or end the interview early, feel free to let me know.

**Confidentiality:**

We take the issue of confidentiality seriously. No personal information about you will be shared with anyone outside the study team. We are digitally recording the interview so that we do not lose any details of the discussion, however only the study coordinator and a professional transcriptionist will hear the recording. We ask that you not give your name to preserve confidentiality. Your real name will not appear anywhere in the written transcripts of, or reports concerning, your interview. Any information from the interview that can identify who you are will be changed in any reports or publications coming from this study. As an additional step to ensure confidentiality, if you wish, we can provide you with your transcript to review and remove any further information you feel may be identifying. Please be assured that the information provided by you will be kept strictly confidential. While all safeguards are in place to maintain their privacy and confidentiality, there is always a risk associated with transfer of data electronically. We hope you will feel comfortable to speak freely. We genuinely want to hear your perceptions. The interview will likely last approximately 10 to 20 minutes.

Do you have any questions about the study details I have just described?

I will now turn on the audio recorder and ask you a few questions to formally record your consent.

Have you heard all of the study details?

Have all of your questions been adequately answered?

Do you agree to be interviewed for research purposes? Do you have any questions or concerns about the process? If not, let’s begin….

***Section 1: Semi-Structured Questions***

1. I would like you to think back to a time when you were involved in healthcare decision making involving [you/your family members/relatives/friends] care (e.g., decisions about tests and/or treatments, use of medication) ?
   1. How were you involved?
2. In an ideal world, how would you like to have been involved in any healthcare decisions?
   1. When?
   2. What types?
   3. Why or why not?
3. Choosing Wisely is an international campaign to engage patients and physicians in discussions to support patients in choosing care that is necessary, free of harm and supported by best evidence. What do you think about the idea of doctors and patients having conversation’s about unnecessary tests and treatments?
   1. How should this be done?
   2. If no, why?
4. How comfortable are you in engaging in discussion with providers about choosing care that is necessary, free of harm and supported by best evidence?
   1. Why?
   2. How would you feel if a doctor spoke to you about this?
   3. How would you feel about raising it with a doctor?
5. What are the benefits of including patients (family members, caregivers, relatives, friends etc.) or members of the public?
6. What are the risks of including patients (family members, caregivers, relatives, friends etc.) or members of the public?
7. What would you need to feel comfortable in engaging in these conversations?
   1. Documents
   2. Training
   3. What would these look like?
8. Is there anything else that I have missed that you would like to add?

Are you interested in reviewing your de-identified transcript and any de-identified quotes that we may choose to use in final reports from this study?

(If yes) Through what mode shall we send you the de-identified data?

- Email address
- Home mailing address (regular mail)

Thank you for sharing your insights with me. If you have no further insights to share, I will now turn off the recorder and ask you some demographic questions.

Interview Guide – Society member involved in list creation

**Preamble:**

Thank you for agreeing to speak with us today. This study is about understanding the role of patients and family members in healthcare decision making. We have asked you to take part in this interview because you have experience in the development of your society’s Choosing Wisely Things Patients and Physicians Should Question List.

This interview will focus on your previous involvement in the development of the society list and your personal perceptions on the current and future role of patients in the Choosing Wisely campaign. Our primary focus is to explore what role patients and family members played in the development and implementation of “Things Physicians and Patients Should Question” Choosing Wisely list, should they be involved, and if so how. We have a series of questions we will ask you. These topics serve as a guide only. If there are other insights you would like to offer, we would like to hear them. We will also ask you a few brief demographic questions. We look forward to the opportunity to learn from your insights.

This interview is completely voluntary. If at any point you feel uncomfortable with the process and wish to end the call, you are free to do so. We recognize that this might be a sensitive topic for you and we want to make you feel as comfortable as possible. If you feel discomfort and would like to skip a question or end the interview early, feel free to let me know.

**Confidentiality:**

We take the issue of confidentiality seriously. No personal information about you will be shared with anyone outside the study team. We are digitally recording the interview so that we do not lose any details of the discussion, however only the study coordinator (Chloe de Grood) and a professional transcriptionist will hear the recording. We ask that you not give your name to preserve confidentiality. Your real name will not appear anywhere in the written transcripts of, or reports concerning, your interview. Any information from the interview that can identify who you are will be changed in any reports or publications coming from this study. As an additional step to ensure confidentiality, if you wish, we can provide you with your transcript to review and remove any further information you feel may be identifying. Please be assured that the information you provided will be kept strictly confidential. While all safeguards are in place to maintain their privacy and confidentiality, there is always a risk associated with transfer of data electronically. We hope you will feel comfortable to speak freely. We genuinely want to hear your perceptions. The interview will likely last approximately 10 to 20 minutes.

Do you have any questions about the study details I have just described?

I will now turn on the audio recorder and ask you a few questions to formally record your consent.

Have you heard all of the study details?

Have all of your questions been adequately answered?

Do you agree to be interviewed for research purposes? Do you have any questions or concerns about the process? If not, let’s begin….

***Section 1: Semi-Structured Questions***

1. What do you think about the idea of doctors and patients having conversation’s about unnecessary tests and treatments?
   1. How should this be done?
   2. If no, why?
2. I would like you to please think back to the process of creating your society’s “Things Patients and Physicians Should Question” list. Please describe this process to me.
   1. Who was involved?
   2. What role did individuals perform?
   3. How did the process work?
   4. What worked well? What could have worked better?
3. In an ideal world, what is the role of patients (family members, caregivers, relatives, friends etc.) or members of the public to be involved in this process?
   1. Why?
   2. How?
4. How comfortable are you in engaging patients or members of the public in choosing care that is necessary, free of harm and supported by best evidence?
   1. Why?
5. What are the benefits of including patients (family members, caregivers, relatives, friends etc.) or members of the public?
6. What are the risks of including patients (family members, caregivers, relatives, friends etc.) or members of the public
7. What would you need to feel comfortable in engaging them in these conversations?
   1. Documents
   2. Training
   3. What would these look like?
8. Is there anything else that I have missed that you would like to add?
9. Is there anyone else you can think of who would be interested speaking to me on this topic – specifically I am interested in talking to members of societies and patients, family members?

Are you interested in reviewing your de-identified transcript and any de-identified quotes that we may choose to use in final reports from this study?

(If yes) Through what mode shall we send you the de-identified data?

- Email address
- Home mailing address (regular mail)

Thank you for sharing your insights with me. If you have no further insights to share, I will now turn off the recorder and ask you some demographic questions.
