# Supplementary material for: Patient and family involvement in Choosing Wisely initiatives: a mixed methods study
Source: BMC Health Serv Res. 2022 Apr 7;22:457. doi: 10.1186/s12913-022-07861-2 (PMC8991491; doi:10.1186/s12913-022-07861-2)
Supplement: Supplementary file 6 — Additional file 6. Structured Questions. Structured questions from qualitative interviews. [file 12913_2022_7861_MOESM6_ESM.docx]

Additional File 6 – Structured Questions

***Structured Questions***

We are collecting personal and family demographic information in order to describe our participants in aggregate and to determine whether they are associated with different experiences of healthcare decision making critical care medicine. Contact information is only for us if you would like to review the report generated from this work to ensure that it reflects your experiences. Please note that your demographic information and contact info will be stored in a password protected database that is only accessible to the study research team. If you are not comfortable answering any of the below questions you are welcome to skip any or all of those you do not wish to answer.

If applicable: What email address/mailing address do you wish to receive your transcript?

______________________________________________________________________________

1. What is your profession?

______________________________________________________________________________

1. What is your role with your society?
2. What is your sex?

⃝ Male ⃝ Female

1. What year were you born in?

______________________________________________________________________________

1. What is your primary language spoken at home?
