# Supplementary material for: Patient and family involvement in Choosing Wisely initiatives: a mixed methods study
Source: BMC Health Serv Res. 2022 Apr 7;22:457. doi: 10.1186/s12913-022-07861-2 (PMC8991491; doi:10.1186/s12913-022-07861-2)
Supplement: Supplementary file 7 — Additional file 7. Detailed Description of Data Analysis. Detailed description of data analysis for qualitative interviews. [file 12913_2022_7861_MOESM7_ESM.docx]

Additional File 7 – Detailed Description of Data Analysis

All transcripts were read in their entirety independently by two researchers (CD and ES) to familiarize with the data, 2) Preliminary coding of 5 transcripts from both patient and professional society representative participant groups occurred independently by the two researchers, 3)The two researchers met to reconcile their coding of the data and organization of emergent themes, 4) During this meeting both researchers developed the initial coding template, 5) Next the coding template was applied independently and in duplicate to an additional 5 transcripts. Researchers met again to discuss necessary modifications to the template to capture data that was relevant to the research question by collapsing larger concepts and defining new themes that were distinct from the previous version of the template. 6) After this meeting an updated version of the template was created and applied to the remaining transcripts. Following this, researchers met to refine and create a final template at which point all transcripts were re-examined under the newest version of the template to further organize and update the themes [Additional File 8].
