# Supplementary material for: Patient and family involvement in Choosing Wisely initiatives: a mixed methods study
Source: BMC Health Serv Res. 2022 Apr 7;22:457. doi: 10.1186/s12913-022-07861-2 (PMC8991491; doi:10.1186/s12913-022-07861-2)
Supplement: Supplementary file 8 — Additional file 8. Final Template. [file 12913_2022_7861_MOESM8_ESM.docx]

Additional File 8 – Final Template

1. Impact of perceived power dynamics on the discussion of low-value care in the clinical interaction

1.1 Assumed roles of patient and care provider

1.2 Individualized context of care decisions

1.3 Physical Presence

1.4 Care providers should be trained to engaged family’s discussions about low-value care

1. How to communicate about low-value care
   1. Understanding the factors that contribute to decisions around low-value care
   2. Communication preferences and strategy different for each family
   3. Empowering patients and families on how to communicate
   4. Care provider suggestions on decision making
2. Perceived barriers to patient involvement in reducing low-value care

3.1 Brief clinical interaction

3.2 Societal Assumptions

3.3 Family and caregivers may not be aware of potential role in decision making

3.4 Tokenism

3.5 Broad nature of topics covered by clinician lists

4. Suggested strategies to engage patients and families in Choosing Wisely initiatives

4.1 Conversations about low-value care centered around care interaction

4.2 Consistency in patient engagement

4.3 Multidimensional approach

4.4 Educate patients and families to advance their knowledge of why a test/treatment or procedures isn’t needed

4.5 Deliver input on messaging of recommendations

4.6 Develop patient-clinician partnerships
