# Supplementary material for: Patient and family involvement in Choosing Wisely initiatives: a mixed methods study
Source: BMC Health Serv Res. 2022 Apr 7;22:457. doi: 10.1186/s12913-022-07861-2 (PMC8991491; doi:10.1186/s12913-022-07861-2)
Supplement: Supplementary file 9 — Additional file 9. Characteristics of North American (USA, Canada) Choosing Wisely clinician lists that involved patients and/or family members (n = 6). [file 12913_2022_7861_MOESM9_ESM.docx]

Additional File 9. Characteristics of North American (USA, Canada) Choosing Wisely clinician lists that involved patients and/or family members (n=6).

| **Society** | **Target clinical population** | **Number of patients involved** | **Level of patient engagement*** | **Role** |
| --- | --- | --- | --- | --- |
| American College of Rheumatology | Rheumatology | 6 | Partner | Patients reviewed final clinician list |
| Canadian Association of Gastroenterology | Gastroenterology | 2 | Partner | Patient representatives were member of the working group |
| Canadian Nurses Association | Nursing | Not reported | Partner | Patient advocates reviewed final clinician list |
| Canadian Psychiatric Association | Psychiatry | 1 | Partner | Patient was a member of working group that selected and prioritized recommendations |
| Canadian Rheumatology Association | Rheumatology | Not reported | Partner | Patient collaborators reviewed clinician list for translation to lay language and accessibility to patients and public |
| Long Term Care Medical Directors Association of Canada | Long term care | 1 | Partner | Patient champion was member of working group in reviewing valid and relevant recommendations |

*According to Born et al. framework of Partner (partnerships with patient organizations to develop and implement campaign materials and initiatives), Engage, Inform, Empower. (1)
