# Supplementary material for: Patient and family involvement in Choosing Wisely initiatives: a mixed methods study
Source: BMC Health Serv Res. 2022 Apr 7;22:457. doi: 10.1186/s12913-022-07861-2 (PMC8991491; doi:10.1186/s12913-022-07861-2)
Supplement: Supplementary file 10 — Additional file 10. North American (United States, Canada) Choosing Wisely patient resources focused on reducing low-value care (n = 148). [file 12913_2022_7861_MOESM10_ESM.docx]

Additional File 10. North American (United States, Canada) Choosing Wisely patient resources focused on reducing low-value care (n=148).

| **Characteristic** | **N(%)** |
| --- | --- |
| **Country** |  |
| USA | 113(76) |
| Canada | 35(24) |
| **Framework Patient Engagement*** |  |
| Inform | 94(64) |
| Inform, Activate | 42(28) |
| Inform, Activate, Collaborate | 10(7) |
| Inform, Collaborate | 2(1) |
| **Overall message of patient resource document** |  |
| Circumstantial use | 107(72) |
| Alternative options | 8(5) |
| Both | 33(22) |
| **Defining effect of low-value care** |  |
| Lack of efficacy | 7(4) |
| Risks outweigh benefits | 13(8) |
| Not cost effective | 1(1) |
| Lack of efficacy & risks outweigh benefits | 26(18) |
| Lack of efficacy & not cost effective | 9(6) |
| Not cost effective & risks outweigh benefits | 35(24) |
| Lack efficacy, risks outweigh benefits & not cost effective | 57(39) |
| **Number of languages available** |  |
| English | 44(30) |
| English and Spanish | 69(47) |
| English and French | 35(23) |

*Fiest et al. engagement strategies: Inform, Activate, Collaborate(2)
