# Supplementary material for: Patient and family involvement in Choosing Wisely initiatives: a mixed methods study
Source: BMC Health Serv Res. 2022 Apr 7;22:457. doi: 10.1186/s12913-022-07861-2 (PMC8991491; doi:10.1186/s12913-022-07861-2)
Supplement: Supplementary file 11 — Additional file 11. Interview Participant Characteristics. [file 12913_2022_7861_MOESM11_ESM.docx]

Additional File 11. Interview Participant Characteristics.

| **Characteristic** | **Number of participants (n=17)** |
| --- | --- |
| **Role** | |
| Physician | 7 |
| Family Member | 6 |
| Patient | 3 |
| Nurse | 1 |
| **Sex** | |
| Female | 12 |
| Male | 5 |
| **Year of Birth** | |
| 1950-1959 | 4 |
| 1960-1969 | 3 |
| 1970-1979 | 4 |
| 1980-1989 | 3 |
| 1990-1999 | 1 |
| Not Reported | 2 |
| **Primary Language Spoken at Home** | |
| English | 17 |
