# Supplementary material for: Patient and family involvement in Choosing Wisely initiatives: a mixed methods study
Source: BMC Health Serv Res. 2022 Apr 7;22:457. doi: 10.1186/s12913-022-07861-2 (PMC8991491; doi:10.1186/s12913-022-07861-2)
Supplement: Supplementary file 12 — Additional file 12. Patient partner review theme description and supporting free text responses. [file 12913_2022_7861_MOESM12_ESM.docx]

Additional File 12. Patient partner review theme description and supporting free text responses.

| **Theme** | **Exemplar Response** |
| --- | --- |
| **Question 1** |  |
| 1. *Trust in healthcare provider decisions and opinions*   Expectation that healthcare provider is up to date on best evidence regarding clinical practice and will make an appropriate decision based on their expertise regarding test, treatments and procedures | *“If [overuse of urinary catheters and ulcer drugs] is a problem in the hospitals then this information should be strongly presented to physicians and doctors and hospital policy makers. I would trust that my doctor would make the right decisions for me.”* |
| 1. *Lack of information*   Better information needs to be included in the patient resource documents including suggestions for prevention, highlighting the risks of disease, pros and cons of procedures, and general knowledge to improve utility and understanding of low-value care practices. | *“I would need to find out where it fit in the assessment and diagnosis range and what options would be available to me and why one would be preferred over another…. the info says, “If your doctor recommends using transvaginal mesh, ask why.” But it doesn’t say why you should ask that, and it doesn’t have any follow up questions…. So, it feels like someone doesn’t want you to get it and thinks your doctor is wrong, but they don’t say why…. They don’t give any pros and cons for the mesh procedure so why do they tell you to challenge your doctor on the procedure? Every doctor has reasons why they are going to do something so why tell the patient to ask [questions]and then leave them hanging as to why they were supposed to ask”* |
| 1. *Tone and language of documents negatively impact ability to engage with low-value care information*   Tone and language of some of the documents is not well tailored for patients, it is too scary and at a level not accessible to patients or their family members or people without more in-depth medical knowledge. | *“This [patient resource document] scares the crap out of me, and I am not easily frightened by medical information… it makes it sound like doctors don’t know what they are doing… so am I supposed to trust a random pamphlet in a doctor’s office or online as opposed to the recommendation of my physician when he or she finds a skin cancer situation on my body? This one needs to be re-written as to “when specific tests may be ordered for this situation and why they would be useful, what they will not provide……” not undermine the physician’s expertise and confuse the patient…. This brochure makes me question the Choosing Wisely program…. In a bad way”* |
| 1. *Worry of what-ifs and the impacts of avoiding tests or treatments*   While patients and their family members may understand the information around reducing tests that are low-value there is concern that something may be missed that will lead to a bad outcome. | *“I think this is good information but there is still a strong fear of people having bad outcomes because they did not receive a CT scan in the emergency department when they should have. That is concerning to me. I don’t believe that people always exhibit textbook symptoms…doctors and support staff need to be on alert and educate family members of patients to support the follow up observations with this type of educational information”* |
| **Question 2** |  |
| 1. *Need for clarification*   The documents serve as a starting place for a discussion with the healthcare provider particularly when they need more clarity about the Choosing Wisely recommendations or a test/treatment/procedure itself. | *“It [the patient resource document] doesn’t say why some of the tests are not good for babies…. I would ask more about that.”* |
| 1. *Interest in options*   Information provided in the patient resource documents provides a starting place for patients and their family members to have discussions with healthcare providers regarding other potential options in treatment. | *“I probably wouldn’t discuss it in the way they recommend…. [They recommend if your doctor suggests it, ask if it’s really necessary] I would likely ask what the options are and then what the pros and cons are.”* |
| 1. *Tone and language negatively impact intentions to discuss with healthcare providers*   The language in some of the documents do not set up the patient to have a conversation with healthcare providers as it has an uncomfortable tone, is confusing and/or undermines the healthcare provider | *“This leaves me a bit puzzled about laparoscopy… it says its usually not needed but then says how many things it can identify which can be fixed by doctors…. Then it proceeds to says the scary risks…. And by the time you get to when you might need one, it talks about venereal diseases. It is a really uncomfortable information process that would make me uncomfortable with even talking to the doctor. The information could be presented in a more friendly manner and less scare tactics.”* |
| 1. *Knowledge on when and how to address*   Provides guidance on what to discuss with physicians and when it may be necessary to reach out. | *“This information does explain lifestyle changes to manage bowel conditions better and information on the condition so that patients can understand the process of the procedure and know what questions to ask and what things to watch for, so they know when to consult a health professional”* |
| 1. *Timing when patient resource document and is provided*   Intention to discuss with healthcare provider is influenced by the timing of when and where this information is presented as it pertains to educating the patient for potential discussions. | *“The right timing of the information…. If I get this at the emerg dept when my child is not well and we are being sent home, it might help me feel more comfortable with the decision the emerg doctor made but if my older parent is exhibit signs and not behaving normally with their breathing etc. and they decide not to do one and give me this, I would feel like I was getting blown off and being told to take care of it myself because it’s not worthy of their time and cost.”* |
